# Applying for a doctorate in nursing? Things you should avoid from getting your application rejected

**DOI:** 10.1002/nop2.561

**Published:** 2020-07-07

**Authors:** Ahtisham Younas, Parveen Ali

**Affiliations:** ^1^ Faculty of Nursing Memorial University of Newfoundland & DSW, Momentum Development Support St John's NL Canada; ^2^ Health Sciences School Division of Nursing and Midwifery The University of Sheffield Sheffield UK

Doctorally prepared nurse scientists play an essential role in creating disciplinary nursing knowledge, developing innovative and patient‐centred interventions, and translating nursing knowledge into practice to improve the standards of nursing care (Broome & Fairman, 2018; Younas & Porr, 2019). Concerns have been raised that existing experienced nurse scientists are at the age of retirement, and there is global shortage of nurses in doctoral programmes (Buerhaus, Skinner, Auerbach, & Staiger, 2017; McSweeney, Weglicki, & García, 2018). There have been calls to action to promote the entry of nurses into doctoral programmes (Broome & Fairman, 2018; McSweeney et al., 2018). However, nurses noted barriers such as time, money, work–life balance, family commitments and lack of motivation to pursue doctoral education (Cavanagh & Alshehry, 2016; Gorczyca, 2013; Squires, Kovner, Faridaben, & Chyun, 2014). Amongst these barriers, one of the common barriers is limited knowledge about the application process, limited confidence in preparing a high‐quality graduate application and fear of rejection (Gorczyca, 2013). We aim to highlight six factors that may negatively affect nurses' confidence to prepare excellent doctoral applications and lead to rejection.

## 1 OVEREMPHASIZING UNIVERSITY RANKING AND DISREGARDING PROGRAMME OF RESEARCH

Choosing a university for a doctoral programme based on *Times Higher Education* and *Quacquarelli Symonds* (QS) university rankings has become an obsession in the current academic world. Some students merely want to get admission into highly ranked universities without considering the nature, depth and type of research programmes offered at the universities. This could be attributed to the notion that getting a doctorate from highly ranked universities would enable them to get an academic position and become recognized expert in their research fields. Highly ranked universities may help to achieve this aim to some extent, but equally important is the alignment of your intended programme of research and the university's offered programmes. Therefore, it is important that a university is chosen based on its research programmes, research environment and the research programme of potential doctoral mentors.

## 2 SKIMMING THROUGH THE PROFILE OF THE POTENTIAL MENTOR

Merely skimming through the research profile of your potential mentor without gaining greater insights into the mentor's research work is detrimental to your first impression as a graduate student. In this case, your email indicating that you are interested in working with the mentor would end up in the trash. Mentors are interested in candidates who are responsible, keen readers and learners, and well‐versed about the mentor's programme of research so that they can mentor you to become excellent scientists. If you decide to ignore in‐depth reading of your potential mentor's work, it might come across that you lack the analytical research skills required for successful and quality doctoral research.

## 3 SENDING SIMILAR APPLICATION TO MULTIPLE MENTORS OR UNIVERSITIES

Some students may be tempted to send one application to various mentors and universities without tailoring it to meet the university requirements and matching it to the mentor's research work. Perhaps, some may send the same application to two or more mentors in the same university. Such actions may show that you are not devoted to putting in some extra efforts and looking for loopholes and shortcuts. Compounding this, if the mentors who received the same application communicated to each other, your prospects of becoming a doctoral student at that university are even slimmer. Therefore, it is essential to submit effective, distinct and well‐tailored applications based on your knowledge of the programme of research of the chosen university and the mentor.

## 4 OVER RELYING ON YOUR ACADEMIC GRADES FOR SUCCESS

Almost every university has set a minimum level of grade and test scores for admission into their graduate programmes. The grades and test scores are one of the factors that strengthen your application. However, it is important that factors such as your personal and professional goals; your chosen programme of research and its alignment with the university's mission and research programmes; and your skills (i.e. time management, research, interpersonal, conflict management) to successfully complete your doctorate are not overlooked. Academic grades are reflective of your academic skills, but may not be good indicators of your interpersonal and problem‐solving skills.

## 5 DOWNPLAYING THE ROLE OF MOTIVATION AND RECOMMENDATION LETTERS

The motivation letter/personal statement and the letters of recommendation from your previous mentors, employers or colleagues are required for doctoral application across all the universities. Unrealistic goals and ambitions statements, unwarranted demonstrations of personal and professional traits, and mismatch of the content of motivation and recommendation letters could lead to application rejection. The recommendation letters which provide no information about your potentials and limitations as a future scientist and offer mere praise of your grades are not useful. Therefore, be wise in choosing the professionals for writing your recommendation letters and offer a realistic overview of your motivation to pursue doctoral studies.

## 6 SUBMITTING A VAGUE AND UNIMPORTANT RESEARCH PROPOSAL

Research proposals are always required as part of a graduate application. Universities and mentors use these proposals to evaluate your research plans and their coherence with the university's and the mentor's research work. However, the research proposals are used to assess skills such as logic, scholarly writing, argumentation and research knowledge, which are essential for successful doctoral education. Submitting vague, overly ambitious, unstructured and unimportant research proposals that may not add anything new to the disciplinary nursing knowledge are often disadvantageous to your application. Even if you have an excellent profile, potential and recommendation to pursue doctoral studies, the inability to justify the significance of your research may result in rejection of your application. A good strategy is to structure your research proposal like an extended structured abstract of the kind you see in the leading journals. Therefore, make provision—using sub‐headings—for an aim, a background, a design and methods. This makes your proposal easy to read and it shows you are capable of thinking logically.

In conclusion, preparing a successful graduate application is daunting. Getting applications rejected could demotivate you from pursuing a doctoral degree in nursing. Some of the suggestions offered in this editorial could be useful for preparing excellent applications. The nursing profession needs more doctoral prepared scientists (Broome & Fairman, 2018; McSweeney et al., 2018), and a successful doctoral application is a prerequisite to become a nurse scientist and contribute towards the development of nursing as a research discipline.

## CONFLICT OF INTEREST

Dr. Parveen Ali is the Editor of Nursing Open.

## References

[nop2561-bib-0001] Broome, M. E. , & Fairman, J. (2018). Changing the conversation about doctoral education in nursing. Nursing Outlook, 66(3), 217–218. 10.1016/j.outlook.2018.04.011 29887188

[nop2561-bib-0002] Buerhaus, P. I. , Skinner, L. E. , Auerbach, D. I. , & Staiger, D. O. (2017). Four challenges facing the nursing workforce in the United States. Journal of Nursing Regulation, 8(2), 40–46. 10.1016/S2155-8256(17)30097-2

[nop2561-bib-0003] Cavanagh, S. J. , & Alshehry, A. (2016). Motivations and barriers for Saudi nurses to pursue a doctoral degree. Retrieved from https://sigma.nursingrepository.org/handle/10755/616053

[nop2561-bib-0004] Gorczyca, C. (2013). Factors influencing the pursuit of graduate education in registered nurses: Exploring the motivators and barriers (Doctoral dissertation, University of British Columbia). Retrieved from https://www.semanticscholar.org/paper/Factors‐influencing‐the‐pursuit‐of‐graduate‐in‐%3A‐Gorczyca/cab2eba0ad627e2201a1a3a1ba60056b53823b38

[nop2561-bib-0005] McSweeney, J. C. , Weglicki, L. S. , & García, R. (2018). Are nursing scientists an endangered species? Journal of Professional Nursing, 34(6), 429–430. 10.1016/j.profnurs.2018.10.005 30527688

[nop2561-bib-0006] Squires, A. , Kovner, C. , Faridaben, F. , & Chyun, D. (2014). Assessing nursing student intent for PHD study. Nurse Education Today, 34(11), 1405–1410. 10.1016/j.nedt.2013.09.004 24080267

[nop2561-bib-0007] Younas, A. , & Porr, C. (2019). Advancing knowledge translation: Is there a role for doctoral students? Journal of Advanced Nursing, 75(5), 924–926. 10.1111/jan.13966 30734360

